# Real-World Experience Treating Pediatric Epilepsy Patients With Cenobamate

**DOI:** 10.3389/fneur.2022.950171

**Published:** 2022-07-12

**Authors:** Konstantin L. Makridis, Thomas Bast, Christine Prager, Tatjana Kovacevic-Preradovic, Petra Bittigau, Thomas Mayer, Eva Breuer, Angela M. Kaindl

**Affiliations:** ^1^Department of Pediatric Neurology, Charité–Universitätsmedizin Berlin, Berlin, Germany; ^2^Center for Chronically Sick Children, Charité–Universitätsmedizin Berlin, Berlin, Germany; ^3^German Epilepsy Center for Children and Adolescents, Charité-Universitätsmedizin Berlin, Berlin, Germany; ^4^Institute of Cell- and Neurobiology, Charité–Universitätsmedizin Berlin, Berlin, Germany; ^5^Epilepsiezentrum Kork, Diakonie Kork, Kehl, Germany; ^6^Epilepsiezentrum Kleinwachau gemeinnützige GmbH, Radeberg, Germany; ^7^Epilepsie-Zentrum Berlin-Brandenburg, Ev. Krankenhaus Königin Elisabeth Herzberge, Berlin, Germany

**Keywords:** epilepsy, Cenobamate, anti-seizure medication, seizure freedom, outcome, children, pediatrics, adverse effects

## Abstract

**Introduction:**

In one third of all patients with epilepsy, seizure freedom is not achieved through anti-seizure medication (ASM). These patients have an increased risk of earlier death, poorer cognitive development, and reduced quality of life. Cenobamate (CNB) has recently been approved as a promising novel ASM drug for the treatment of adults with focal-onset epilepsy. However, there is little experience for its application in pediatric patients.

**Methods:**

In a multicenter study we evaluated retrospectively the outcome of 16 pediatric patients treated “off label” with CNB.

**Results:**

In 16 patients with a mean age of 15.38 years, CNB was started at an age of 15.05 years due to DRE. Prior to initiation of therapy, an average of 10.56 (range 3–20) ASM were prescribed. At initiation, patients were taking 2.63 (range 1–4) ASM. CNB was increased by 0.47 ± 0.27mg/kg/d every 2 weeks with a mean maximum dosage of 3.1 mg/kg/d (range 0.89–7) and total daily dose of 182.81 mg (range 50–400 mg). Seizure freedom was achieved in 31.3% and a significant seizure reduction of >50% in 37.5%. Adverse events occurred in 10 patients with fatigue/somnolence as the most common. CNB is taken with high adherence in all but three patients with a median follow-up of 168.5 days

**Conclusion:**

Cenobamate is an effective ASM for pediatric patients suffering from drug-resistant epilepsy. In addition to excellent seizure reduction or freedom, it is well-tolerated. Cenobamate should be considered as a novel treatment for DRE in pediatric patients.

## Introduction

Epilepsy is one of the most common neurological disorders in children (1). Epilepsy is drug-resistant in about one third of the pediatric epilepsy population, i.e., treatment with two or more correctly chosen and dosed anti-seizure medications (ASMs) does not achieve seizure freedom (2). In these patients, the chance of reaching seizure freedom is lower than 15% (3). Given the increased risk of earlier death, poorer cognitive development, and reduced quality of life of patients with drug resistant epilepsy (DRE), (4, 5) the possibility of a curative approach through epilepsy surgery must be assessed early (6, 7). However, even in individuals with focal-onset DRE, epilepsy surgery is not always feasible as a curative (or palliative) approach. In these patients, further ASM treatment is needed.

Cenobamate (CNB) is a new ASM recently approved by the US Food and Drug Administration and the European Medicines Agency for the treatment of focal-onset seizures in adults. While the exact mode of action of CNB is not known, it has been shown to enhance inhibitory currents at GABA_A_ receptors (8, 9) and to block excitatory currents by promoting the inactivated state of voltage-gated sodium channels (10).

Results regarding CNB efficacy are promising with placebo-adjustment seizure freedom in ~20% and seizure reduction of >50% in about half of patients with focal seizures who were concomitantly taking up to three ASM (11). CNB was taken with high retention, suggesting good long-term tolerability (12). CNB is not approved for use in children and adolescents despite the high need for treatment in the pediatric population. Hence, there is no experience for use of CNB in the pediatric epilepsy population with respect to dosing, side-effects, and efficiency. Here we describe our experience with CNB in 16 pediatric patients.

## Methods

We performed a retrospective multicenter study of pediatric patients treated with CNB. All patients up to 18 years of age at initiation of treatment were included. All patients being treated with CNB at the epilepsy centers participating in this multicenter study were included in this study. Data was extracted from medical files using a standardized data sheet. Data on seizure reduction and side effects are based on information provided by parents and patients. For the evaluation of therapy success, seizure frequency was compared to the 4 weeks before therapy initiation and the last 2 weeks under therapy when therapy duration was <3 months. For patients with longer therapy duration, the last 4 weeks were compared. The cumulative data was then imported into SPSS 28 for analysis and evaluation. Descriptive statistics were used to calculate frequencies and percentages. Group data are presented as mean ± standard deviation unless otherwise stated. The study was approved by the local ethics committee (approval no. EA2/084/18).

## Results

A total of 16 pediatric patients with DRE (56.3% female, 43.8% male) were treated with CNB at a mean age of 15.05 ± 1.64 years (range 12.08–17.67) at treatment start (Table 1). The median age at seizure onset was 39 months-of-age (range 0–108) and the average disease duration was 11.73 years (range 6.33–16.42). Epilepsy causes included abnormalities of cortical development (*n* = 5), residual brain damage following infarction, asphyxia or encephalitis (*n* = 4), genetic diseases (*n* = 2) and autoimmune (*n* = 1). The epilepsy cause was unclear in 25% of cases. At initiation of CNB the patients were prescribed concomitantly a mean number of 2.63 ASM (range 1–4), and had a life-time number of 10.56 ASM (range 3–20). The most common ASM were clobazam (*n* = 8), lacosamide (*n* = 6), and lamotrigine (*n* = 5).

**Table 1:** Pediatric patients treated with Cenobamate.

**ID**	**Sex**	**Age at seizure onset (months)**	**Cause**	**Age at CNB start (years)**	**No. of ASM at CNB start (ASM)**	**No. of ASM at CNB start**	**CNB titration protocol starting dosage (total, mg/kg/d); delta per 2 weeks (total, mg/kg/d); maximum dosage (total, mg/kg/d)**	**Outcome**	**Follow up (days)**	**Adverse effects**	**Reduced ASM**	**Discontinued ASM**
1	m	86	FCD	17.67	15	3, CLB, PER, ESL	12.50, 0.12; 33.33, 0.31; 200, 1.87	Free	314%	None	-	-
2	m	48	Unclear	16	12	4, LCM, CLB, CBD, PER	12.500, 15; 25.00, 0.30; 100, 1.2	<50	292%	Somnolence	-	-
3	m	0	Infarction	15.08	6	3, LCM, CLB, BRV	6.25, 0.36; 6.82, 0.1; 75, 1.07	Free	268%	None	-	-
4	m	64	Unclear	12.25	13	2, VPA, OXC	12.50, 0.22; 42.86, 0.74; 300, 5.17	Free	208%	Somnolence, transient seizure increase	-	OCX
5	f	106	Unclear	15.08	8	2, LTG, LCM	12.5, 0.18; 53.33, 0.76; 400, 5.71	<50	199%	Somnolence, vertigo, diplopia, impulsive behavior	LTG	LCM
6*	m	24	Variants LANC3, ALDH7A1, SLC19A3, SZT2 and KCNB1	17	7	3, LCM, BRV, RFM	12.50, 0.13; 33.33, 0.34; 200, 2.03	<50	197%	Increased feeling of hunger, weight gain, poor sleep	-	-
7	w	61	FCD	14.17	11	1, ESL	12.50, 0.25; 43.75, 0.88; 350, 7	>50	193%	None	ESL	-
8	f	93	Unclear	16	10	2, LTG, PGB	12.50, 0.23; 46.15, 0.83; 300, 5.42	>50	180%	Somnolence, impulsive behavior	LTG	PRG
9	f	89	Polymicrogyria	14.08	9	3, LTG, CBD, PER	12.50, 0.29; 36.36, 0.84; 200, 4.6	<50	157%	Impulsive behavior	-	CBD
10	f	4	FCD	14.79	20	3, LEV, CLB, RFM	12.50, 0.20; 10.53, 0.17; 100, 1.61	>50	151%	Eczema	LEV, RFM	-
11	m	30	del 15q11.2, suspected FCD	13.83	20	4, LTG, VPA, CLB, ESL	12.50, 0.21; 25, 0.42; 200, 3.39	>50	150%	Vertigo	LTG, CLB	VPA, ESL
12	f	5	Infarction	14.83	6	3 LTG, VPA, CLB	6.25, 0.18; 9.09, 0.26; 100, 2.89	>50	125%	None	-	-
13*	f	14	Herpes simplex encephalitis	17.58	7	1, LCM	12.5, 0.34; 25, 0.68; 100, 2.73	Free	107%	Nausea, balance disorder, vertigo	LCM	-
14	m	108	Autoimmun	16	3	2, LCM, BRV	25, 0.32; 33.33, 0.42; 200, 2.53	Free	94%	None	-	-
15	f	0	Asphyxia	14.33	12	3, CLB, RFM, CBD	6.25, 0.20; 10, 0.31; 50, 1.56	>50	81%	None	-	-
16*	f	6	FCD	12.08	10	3, CLB, BRV, CBD	12.5, 0.22; 12.50, 0.22; 50, 0.89	Increase	56%	Seizure increase, somnolence	-	-

*f, female; m, male; ASM, anti-seizure medication; CNB, cenobamate; VPA, Valproate; LEV, Levetiracetam; LTG, Lamotrigine; LCM, Lacosamide; OXC, Oxcarbazepine; CLB, Clobazame; BRV, Brivaracetam; RFM, Rufinamide; CBD, Cannabidiol; PER, Perampanel; ESL, Eslicarbazepine acetate; PRG, Pregabalin; -, no*.

^*^Patients in whom CNB was discontinued.

Two individuals had not become seizure free despite epilepsy surgery (P3: lesionectomy; P10 lesionectomy followed by hemispherotomy), and five despite ketogenic diet.

**Figure 1:**
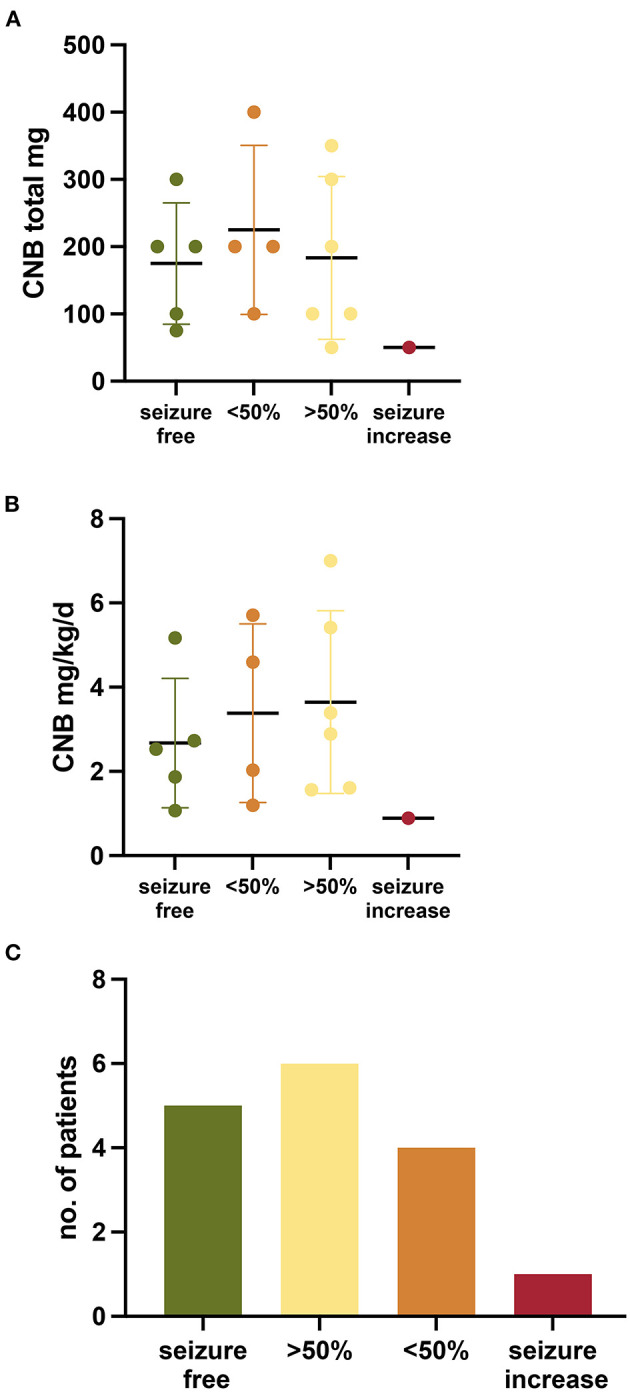
Outcome of patients treated with Cenobamate. **(A,B)** Graph showing the outcome per patient and maximum CNB dosage (mg/kg/d and total) **(C)** Bar chart depicting the outcome of patients treated with Cenobamate.

The titration scheme applied to the cohort varied given that there is no experience in CNB titration in pediatric patients. CNB was given once a day orally. The initial dose chosen in pediatric patients was most frequently 12.5 mg (range 6.25–25) and a mean of 0.22 mg/kg/d (range 0.12–0.36) (Table 1). The mean body weight of individuals was 62.16 kg (range 32–107). In most individuals CNB was increased by 0.47 ± 0.27 mg/kg/d every 2 weeks (27.9 mg ± 14.83). The mean maximum CNB dosage was 3.1 mg/kg/d (range 0.89–7) with a total daily dose of 182.81 mg (range 50–400 mg) (Figures 1A,B). The individual titration until highest dosage is given for each patient in Supplementary Figure 1. In most cases, the dosages of other ASM were kept constant at CNB initiation. In eight patients, other ASMs could even be reduced or discontinued after CNB initiation (4, 5, 7–11, 13). In most cases, ASMs were reduced or discontinued to reduce drug burden, as well as to reduce side effects due to possible pharmacodynamic interaction.

Adverse events (AE) occurred in ten cases (62.5%) during 198 CNB up-titration. However, AE were in most cases no severe enough to result in a discontinuation of CNB. In patient 16 CNB dosing resulted in an increase in seizure frequency and 201 prolongation of seizures. For this reason, CNB was discontinued 8 weeks after initiation, at a dose of 50 mg (0.89 mg/kg/d), under which seizure frequency and duration returned to previous levels. In another patient (Patient 4), a transient increase in seizure 205 frequency occurred. However, the up-titration was continued to a dose of 300 mg (5.17 mg/kg/d). This resulted in seizure freedom. In two other patients, AEs were too severe during the follow-up, and CNB was discontinued (P6, P13). In three patients AEs led to a reduction of dosage (P4, P5, P9). Most common adverse effects were somnolence/fatigue (*n* = 5). In two patients, seizure frequency increased during the titration period as delineated above, leading to a discontinuation of CNB in one patient. Three patients reported agitated behavior. Vertigo was observed in three patients. One patient each reported nausea and a balance disorder, diplopia and increased impulsive and agitated behavior, increased appetite resulting in weight gain and impaired sleep quality. One patient reported a rash with scaling first of the hands, then feet, as well as transient red eczema on the hips, which did not lead to discontinuation of CNB. No drug rash with eosinophilia and systemic symptoms (DRESS) occurred.

The effect of CNB treatment was evaluated at a median follow up of 168.5 days (range 56–314). All patients continued to take CNB except for three (81.25%) Seizure freedom was observed in five patients (31.3%) (Figure 1C). A seizure reduction >50% was observed in six patients (37.5%), four patients (25%) had a reduction of seizures <50%, and one case had an increase of seizure frequency.

## Discussion

Here we report our experience with CNB in 16 pediatric patients with DRE. CNB was initiated in pediatric patients with body weights between 32 and 107 kg most often at 12.5 mg once a day (0.22 mg/kg/d) and then titrated-up by 0.47 ± 0.27 mg/kg/d every 2 weeks. The individual maximum daily dose varied between 50 and 400 mg, with a mean of about 183 mg. Treatment with CNB resulted in seizure-free or a significant seizure reduction of > 50% in more than two thirds of the patients. These rates of seizure freedom or strong reduction of seizure frequency are in line with data published for adults. No serious adverse events occurred in our cohort. AEs occurred in about two thirds of the pediatric cohort, similar to the rate in adults (50%) (12). Two patients had an increase in seizure frequency, which was transient in one of them, and led to a treatment stop in the second patient. Most frequently, somnolence/fatigue occurred during up-titration, in line with the report of Sperling et al. (12). Less frequently vertigo, nausea, balance disorder, diplopia, increased impulsive/agitated behavior, increased appetite with weight gain and impaired sleep quality were reported. No drug rash with eosinophilia and systemic symptoms (DRESS) occurred. However, with a median follow-up of 168.5 days (range 56–314), these data are only a short time experience. Long-term data on the changes in outcome and complications are needed. Large multi-center prospective studies are necessary to answer these questions.

In conclusion, we report our first experience in treatment of pediatric patients with CNB. CNB showed an excellent effect with respect to seizure control in our small cohort of pediatric patients with DRE. The drug was well-tolerated without severe side effects. The use of CNB should, therefore, be considered in pediatric patients with DRE. Still, data for application of CNB in children prior to the age of 12 years and with a body weight below 32 kg are lacking. Furthermore, these data are only a short time experience due to short follow up duration. Given the need of therapeutic approached in children with DRE, large cohort, prospective studies are needed to determine the dosing schemes necessary for various weight ranges, safety data and efficacy data for use in children of all age ranges.

## Data Availability Statement

The raw data supporting the conclusions of this article will be made available by the authors, without undue reservation.

## Ethics Statement

The studies involving human participants were reviewed and approved by Charité Universitätsmedizin Berlin. Written informed consent from the participants' legal guardian/next of kin was not required to participate in this study in accordance with the national legislation and the institutional requirements.

## Author Contributions

KM and AK contributed to the conception and design of the study. KM, TB, TK-P, TM, and EB contributed to acquisition of data. KM organized the database analyzed the data, wrote the first draft of the manuscript, and created figures and tables. All authors discussed the results, revised the first draft, and contributed to the final manuscript.

## Funding

The study was supported by the Einstein Stiftung Fellowship through the Günter Endres Fond and the Sonnenfeld-Stiftung.

## Conflict of Interest

TK-P and TM were employed by Epilepsiezentrum Kleinwachau gemeinnützige GmbH. The remaining authors declare that the research was conducted in the absence of any commercial or financial relationships that could be construed as a potential conflict of interest.

## Publisher's Note

All claims expressed in this article are solely those of the authors and do not necessarily represent those of their affiliated organizations, or those of the publisher, the editors and the reviewers. Any product that may be evaluated in this article, or claim that may be made by its manufacturer, is not guaranteed or endorsed by the publisher.
