# Supplementary figures and images for: Real-World Experience Treating Pediatric Epilepsy Patients With Cenobamate
Source: Front Neurol. 2022 Jul 12;13:950171. doi: 10.3389/fneur.2022.950171 (PMC9350548; doi:10.3389/fneur.2022.950171)

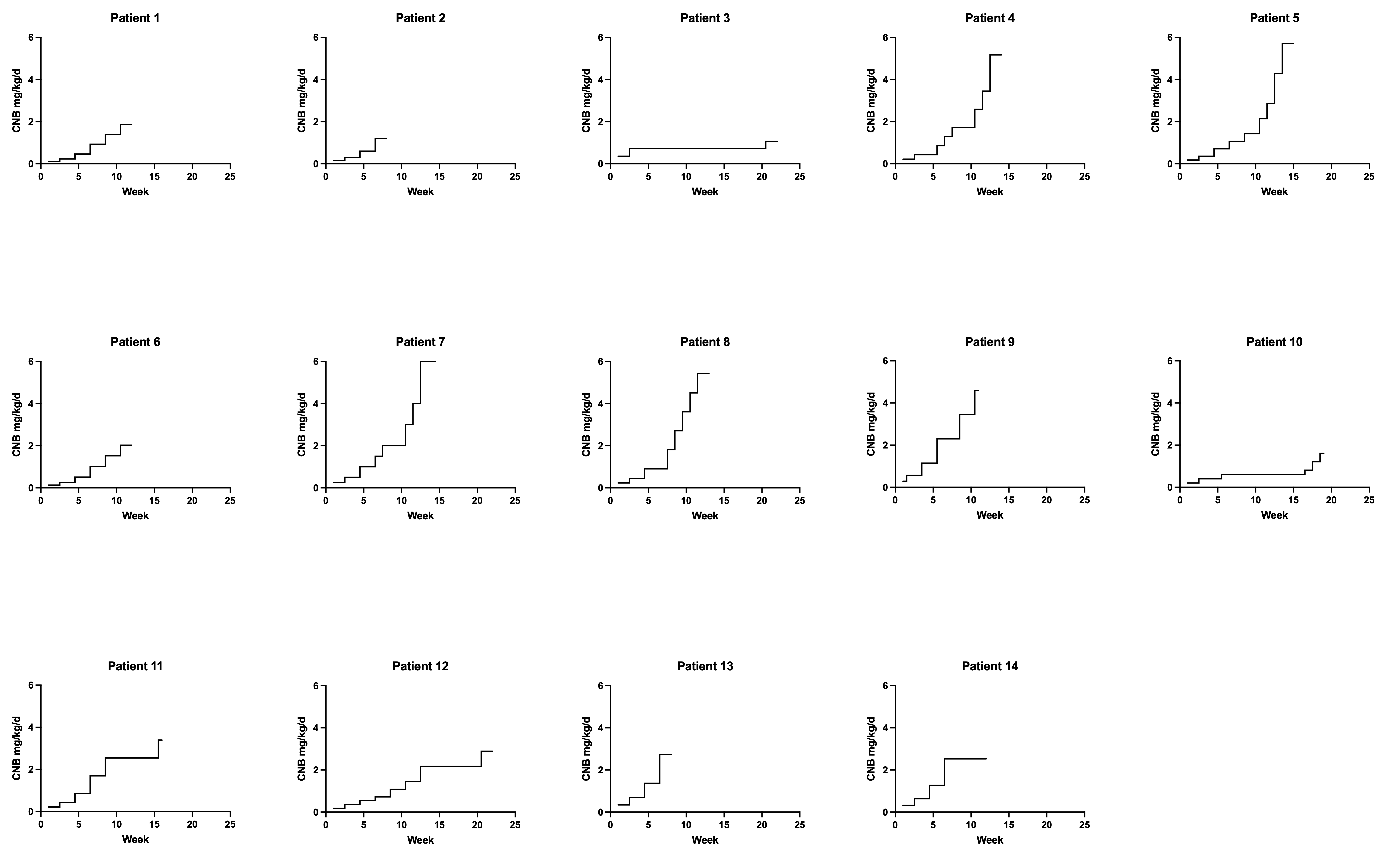

Supplement: Supplementary file 1 [file Image_1.TIFF]
